# The Kesennuma Study in Miyagi, Japan: Study Design and Baseline Profiles of Participants

**DOI:** 10.2188/jea.JE20200599

**Published:** 2022-12-05

**Authors:** Mari Yamashita, Satoshi Seino, Yu Nofuji, Yasuhiro Sugawara, Yosuke Osuka, Akihiko Kitamura, Shoji Shinkai

**Affiliations:** 1 Research Team for Social Participation and Community Health, Tokyo Metropolitan Institute of Gerontology, Tokyo, Japan; 2 Research Team for Promoting Independence and Mental Health, Tokyo Metropolitan Institute of Gerontology, Tokyo, Japan; 3 Undergraduate School of Nutrition Sciences, Kagawa Nutrition University, Saitama, Japan

**Keywords:** frailty, community-dwelling older adults, population approach, Kesennuma Study, psychological and social functions

## Abstract

**Background:**

To clarify the association between psychosocial problems and frailty in the areas affected by the Great East Japan Earthquake, and to develop strategies for preventive long-term care in the community, we launched the Kesennuma Study in 2019. This report describes the study design and the participants’ profiles at baseline.

**Methods:**

The prospective study comprised 9,754 people (4,548 men and 5,206 women) randomly selected from community-dwelling independent adults aged 65 to 84 who were living in Kesennuma City, Miyagi. The baseline survey was conducted in October 2019. It included information on general health, socio-economic status, frailty, lifestyle, psychological factors (eg, personality, depressive moods), and social factors (eg, social isolation, social capital). A follow-up questionnaire survey is planned. Mortality, incident disability, and long-term care insurance certifications will also be collected.

**Results:**

A total of 8,150 questionnaires were returned (83.6% response rate), and 7,845 were included in the analysis (80.4%; mean age 73.6 [standard deviation, 5.5] years; 44.7% male). About 23.5% were considered frail. Regarding psychological and social functions, 42.7% had depressive moods, 29.1% were socially isolated, and only 37.0% participated in social activities at least once a month. However, 82.5% trusted their neighbors.

**Conclusion:**

While local ties were strong, low social activity and poor mental health were revealed as issues in the affected area. Focusing on the association between psychological and social factors and frailty, we aim to delay the need for long-term care for as long as possible, through exercise, nutrition, social participation, and improvement of mental health.

## INTRODUCTION

The Great East Japan Earthquake (GEJE) and tsunami in 2011 led to 15,899 deaths, and 2,528 missing and 6,157 injured people in Japan.^1^ More than 110,000 people had no choice but to live in prefabricated temporary housing for a long period; some continue to live in these conditions. Miyagi Prefecture reported 9,543 deaths and 1,216 missing and 4,145 injured people, making it one of the most severely affected areas. The infrastructure is recovering and no one was living in prefabricated temporary housing as of March 2020.^2^

A previous study assessing the long-term impact of GEJE on older residents reported that disability prevalence increased and sleep problems were more durably linked to material aspects of disaster damage.^3^^–^^6^ Moreover, some older people cannot adapt well to the new community and remain isolated.^7^ Thus, dealing with the psychosocial problems caused by GEJE is an urgent priority, to delay older adults entering long-term care and improve their health.

Frailty involves reduced ability to cope with age-associated stressors; it increases the risks of disability, institutionalization, and mortality.^8^ Frailty has been identified as a pre-stage of independence loss. Preventing frailty helps extend older adults’ healthy life expectancy.^9^ The major independent risk factors for frailty have been classified as lower physical function, lower nutritional status, lower social function and social participation, and preclinical cardiovascular disease.^10^ Psychological factors, such as cognitive decline, depression, and loneliness, have been reported to be associated with frailty.^11^^,^^12^ However, in Japan, the association between psychological and social factors and frailty has not been established. It is necessary to find effective frailty prevention approaches, including psychological aspects, in areas affected by the GEJE.

To identify the associations between psychological and social factors and frailty in older adults according to sex and age, and find effective approaches to delay the need for long-term care in affected areas, we launched the Kesennuma Study in 2019. Here, we describe the study design and participants’ profile at baseline.

## METHODS

### Study design, study setting, and participants

The Kesennuma Study is a participatory action research (PAR) study^13^^,^^14^ based on a prospective cohort study design. The outcomes of the PAR are evaluated using a quasi-experimental design.^15^^,^^16^ Specifically, we aim to assess how psychological and social factors affect frailty through a cohort analysis. Moreover, we consider salons (a general term used in Japan to refer to places older adults can visit to carry out various activities) to be one of the hubs of community-based intervention and will compare frailty status between those who participate in the salon (the intervention group) and those who do not (the control group).

**Figure 1.**
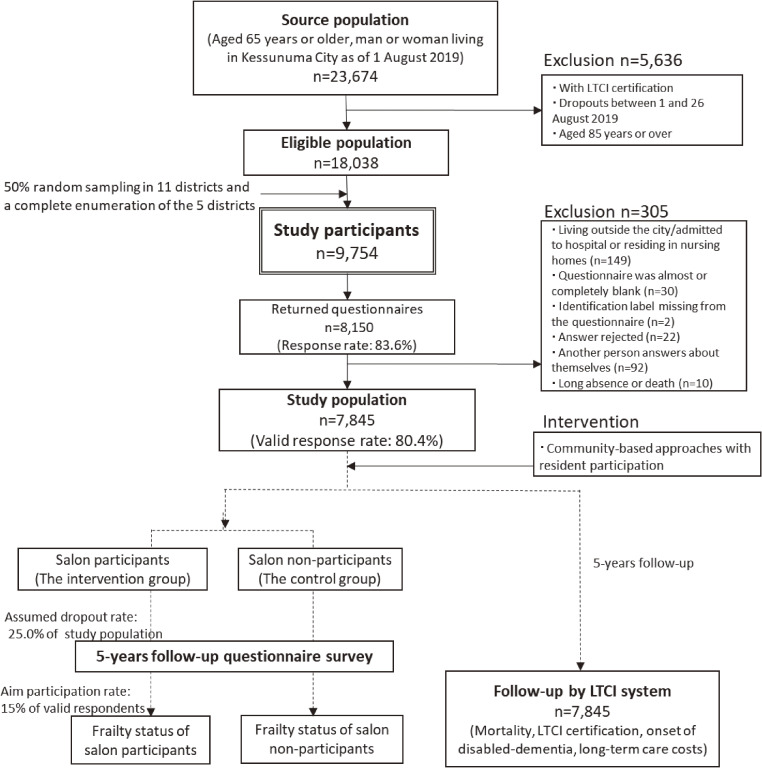
Flow diagram of study participants: the Kesennuma study in 2019

The source population comprised community-dwelling individuals aged 65 or older who lived in Kesennuma City in Miyagi Prefecture, in north-east Japan, one of the GEJE disaster areas. Figure 1 depicts a flow diagram of the study participants. On August 1, 2019, the population aged 65 years or over comprised 23,674 people (10,286 men and 13,388 women). Additionally, the proportion of the population aged 65 and over was 37.1%. Because frailty is a syndrome occurring before falling into a state of needing care, we excluded individuals who were certified for long-term care insurance (LTCI), hospitalized, or living in nursing homes. As we considered it would be a struggle for them to answer many items and return questionnaires by mail, individuals older than 85 years were excluded. The eligible population was 18,038 as of August 26, 2019. For each of Kesennuma Council of Social Welfare’s 16 districts, the survey was conducted using 50% random sampling in 11 large districts and a complete enumeration of the 5 smaller districts (target populations ≤501). Finally, 9,754 people (4,548 men and 5,206 women) were included. To combine the baseline and follow-up survey data, we added identification numbers to questionnaires. The basis for the sample size was calculated as follows. First, Nofuji et al^17^ reported that the prevalence of frailty in participants and non-participants in the frailty prevention class was 17.1% and 24.1%, respectively. To detect this difference of about 7% using a chi-square test, 521 subjects in each of the intervention (salon participants) and non-intervention groups (salon non-participants) were required (statistical power 0.8, risk level 0.05). Second, based on a survey by Nofuji et al,^17^ it was expected that about 25.0% participants at the baseline will drop out 5 years later. Third, considering the baseline survey results, the valid response rate was expected to be 80%. In addition, the target rate for participation to salons, which Kesennuma City is aiming for, was approximately 15.0%. From the above, the number of subjects required for the baseline survey was calculated to be 7,236.

On October 4, 2019, we mailed self-administered questionnaires and requested them to be returned by October 18, 2019. Non-responders were sent postcard reminders and asked to return their questionnaires by November 1, 2019. To increase effective answers, we conducted telephone interview surveys on 830 incomplete questionnaires from November 15 to December 23, 2019.

### Baseline survey

Table 1 summarizes the main measures surveyed at baseline.^10^^,^^18^^–^^20^^,^^23^^–^^37^ The key measures include frailty status (primary outcome) and psychological and social variables (secondary outcomes). Other factors, such as physical function and nutrition status, which have been reported to be related to frailty,^10^ and the effects of the GEJE and socio-economic status were selected as confounding factors (additional measures).

**Table 1.** Outline of baseline self-administered questionnaire on the Kesennuma Study 2019

**Primary outcome measures**	
Frailty	CL15^10^^,^^18^^–^^20^
**Secondary outcome measures**	
Psychological function	Current feeling^23^^–^^26^
	
	Subjective well-being^27^
	Subjective cognitive decline^28^
	Personality traits (TIPI-J)^29^
Social function	Social network^30^
	Frequency of outings^31^
	Cognitive social capital (trust in neighbours)
**Additional measures**	
Demographics	Age, sex
	Years of residence in the neighbourhood
	Living arrangement
Socio-economic status	Education
	Taxation status
	Current job
Medical and lifestyle profiles	Drinking habits, smoking status
	Self-rated health, history of physician-diagnosed diseases
	Body mass index
	Musculoskeletal pain, mobility limitation^32^^,^^33^
	Regular exercise, sitting time^34^
	Dietary information: DVS,^35^^,^^36^ eating alone
	Sleep-related items
	Higher-level competence, TMIG-IC^37^
GEJE-related items	Number of relocations
	Current home
	Change in consultation access

CL15, Check-List 15^10^^,^^18^^–^^20^; DVS, Dietary Variety Score^35^^,^^36^; TIPI-J, Ten Item Personality Inventory^29^; TMIG-IC, Tokyo Metropolitan Institute of Gerontology Index of Competence.^37^

History of physician-diagnosed diseases: hypertension, dyslipidemia, heart disease, stroke, diabetes, bone disease, respiratory organs, cancer.

Sleep-related items: sleep duration, difficulty falling asleep, quality of sleep.

### Primary outcome measures

Frailty status was assessed using a Check-List 15 (CL15) score of ≥4.^10^^,^^18^^–^^20^ The CL15 had three dimensions and 15 items consisting of five items of homeboundness, six items of falling, and four items of lower nutrition (eTable 1). The index score ranges from 0 to 15, with a higher score indicating a greater likelihood of frailty. Shinkai et al^19^ reported that a cut-off score of 3/4 discriminated Fried’s definition frailty^21^ from non-frailty with a sensitivity of 70.0% and specificity of 89.3%. The CL15 is strongly correlated with the Frailty Index,^22^ a significant predictor of LTCI certification and/or mortality, and is compatible with the Frailty Index in predicting risk.^20^

### Secondary outcome measures

Psychological information included current feeling,^23^^–^^26^ subjective well-being,^27^ subjective cognitive decline,^28^ and personality.^29^ Current feeling included depressive moods,^23^^,^^24^ apathy,^25^ and loneliness.^26^ The above indicators have been validated, and other questions were asked about trauma (‘When remembering past sorrows and unpleasant experiences, do you often feel gloomy?’) and anxiety (‘Do you often feel vague uneasiness about the future?’).

Assessed social functions included social network,^30^ frequency of outings,^31^ and cognitive social capital (‘Do you trust your neighbors?’). Social network included social isolation,^30^ social participation (volunteer, sports, and hobby groups, and senior citizen clubs, neighborhood associations, and salon activities), social support, and conversation with others (‘Do you talk to someone every day?’). Social support included availability of instrumental support (‘Can anyone help you when you are in trouble?’), informational support (‘Can anyone give you information and knowledge that you need?’), and emotional support (‘Is there anyone with whom you can share your worries and feelings?’). Face-to-face and non-face-to-face contact with people outside of the household less than 1 day a week is used as an operational definition of social isolation.^30^ The standard of outing frequency that has been shown to be associated with functional decline in the elderly is less than 1 day a week.^31^ Other question items about social network and cognitive social capital have not been validated and are customarily used.

### Additional measures

As additional measures, demographics, socio-economic status, medical and lifestyle profiles, and GEJE-related items were collected. GEJE-related items included number of relocations, current home, and change in consultation access. Answers to change-related question were organized in three groups: positive change (‘Did you have more people to talk to?’), negative change (‘Did you have fewer people to talk to?’/‘I don’t know what to do when I face difficulties’), and unchanged. GEJE-related items were questions decided in consultation with Kesennuma City.

### Intervention overview

Kesennuma City signed a comprehensive cooperation agreement with us in November 2018 to improve regional issues. As such, this study is planned as a joint project to delay the need for long-term care among older residents. First, to understand regional issues and discuss frailty prevention, resident-organized community meetings were held in all 16 districts between July and October 2019. At these community meetings, each district was presented with its results from the baseline survey. Inspired by these findings, residents have begun taking actions to improve issues in their areas. Second, we are planning actions to enhance frailty prevention knowledge levels and behaviors. We educate resident leaders and volunteers in frailty prevention knowledge and implement exercise, nutrition, and social function improvement programmes^17^ for other residents. Furthermore, we plan to use and expand the salon as a place to provide such activities. Finally, we added frailty prevention elements to activities already underway. These steps are based on the PAR framework.^13^^,^^14^

### Follow-up surveys

We plan to conduct a follow-up questionnaire survey 5 years after the baseline survey, which will be mailed to the baseline survey respondents. The follow-up survey will include items related to frailty (primary outcome) and psychological and social factors (secondary outcomes).

In Japan, all individuals aged 65 years or older are eligible for LTCI benefits, and the LTCI system monitors an individual’s LTCI usage via their municipal government. The LTCI classifies individuals as ‘Support Level 1 or 2’ to indicate the need for assistance with basic activities of daily living or ‘Care Level 1 through 5’ to indicate the need for continuous care.^38^ The degree of independence in daily life specified for individuals with dementia is listed separately. The date and time of death or transfer from Kesennuma City are also recorded. In this study, the participants will be tracked monthly regarding the LTCI benefits received for 5 years after the baseline survey, to confirm four outcomes: mortality, LTCI certification, onset of disabled-dementia, and long-term care costs. The LTCI information will be provided to us by Kesennuma City.

### Ethical considerations

The study protocol was approved by the Ethical Committee of the Tokyo Metropolitan Institute of Gerontology (August 25, 2019). All participants gave informed consent. A statement attached to the questionnaire explained the study’s purpose, the voluntary nature of participation, and confirmed the analysis was anonymous. Returning the questionnaire was viewed as consent to participate.

### Statistical analyses

To describe the study participants’ characteristics at baseline, taking into account sex and age, we compared the main measures between participants grouped by age (65–74 and 75–84 years old) and sex (male and female) using the chi-square test for nominal variables, the Kruskal-Wallis test for ordinal variables, and one-way analysis of variance for continuous variables. When comparing Kesennuma City’s characteristics with those of other areas, we used the independent *t*-test for continuous variables and chi-square test for categorical variables. *P*-values under 0.05 on a two-tailed test were regarded as statistically significant. SPSS version 25.0 (IBM Corp., Armonk, NY, USA) was used for statistical analyses.

## RESULTS

Of the 9,754 people surveyed, we collected questionnaires from 8,150 (response rate of 83.6%). A total of 305 people were excluded, including unidentified people, blank entries, peeled-off labels, those clearly answered by another person (non-matching sex and age), hospitalization, refusal to respond, long-term absence, or death. The analysis was conducted on data from 7,845 individuals (valid response rate of 80.4%). Table 2 shows the collection status by the 16 districts.

**Table 2.** The collection status of the 16 districts

District	Target population *n*	Study participants *n* (%)	Returned questionnaires *n* (%)	Study population *n* (%)
A	2,101	1,050 (50.0)	874 (83.2)	842 (80.2)

B	1,253	626 (50.0)	529 (84.5)	511 (81.6)

C	222	222 (100.0)	182 (82.0)	171 (77.0)

D	263	263 (100.0)	218 (82.9)	212 (80.6)

E	134	134 (100.0)	114 (85.1)	111 (82.8)

F	501	501 (100.0)	402 (80.2)	381 (76.0)

G	1,418	709 (50.0)	577 (81.4)	552 (77.9)

H	2,454	1,227 (50.0)	1,041 (84.8)	1,016 (82.8)

I	1,546	773 (50.0)	667 (86.3)	648 (83.8)

J	1,133	566 (50.0)	458 (80.9)	444 (78.4)

K	1,621	810 (50.0)	701 (86.5)	678 (83.7)

L	903	451 (50.0)	366 (81.2)	343 (76.1)

M	1,860	930 (50.0)	790 (84.9)	749 (80.5)

N	357	357 (100.0)	290 (81.2)	279 (78.2)

O	1,269	634 (50.0)	517 (81.5)	495 (78.1)

P	1,003	501 (50.0)	422 (84.2)	413 (82.4)

Total	18,038	9,754 (54.1)	8,148 (83.5)	7,845 (80.4)

Note: Two returned questionnaires lost their identification labels and could not be classified by district.

Table 3 shows participants’ baseline characteristics regarding the additional measures. The mean age was 73.6 (standard deviation, 5.5) years, 44.7% of participants were men, and 94.2% had lived in their community for 30 years or more. Being widowed, living alone, and being unemployed were more frequent in women aged 75 or older. A higher proportion of men had previous or current drinking and smoking habits. The proportion of people who felt healthy and had no chronic disease was higher among people aged 65 to 74 compared to those 75 or older, regardless of sex. Being underweight (body mass index <18.5 kg/m^2^), having musculoskeletal pain, having mobility limitation, and having sleep disorders were more frequent in women aged 75 or older. The proportion of obesity (body mass index ≥25.0 kg/m^2^) was inversely associated with being a man aged 65 to 74. Dietary variety scores and Tokyo Metropolitan Institute of Gerontology Index of Competence (TMIG-IC) scores were higher among women in both age groups. After GEJE, 30.7% had relocated at least once. Relocating three or more times after GEJE was more frequent in women aged 75 or older than other groups. Regarding changes in post-GEJE consultation access, 66.1% of people answered that there was no change, although people aged 75 or over felt more negative changes.

**Table 3.** Participants’ baseline characteristics: additional measures

		Total		Men				Women				*P*
				65–74 years old		75–84 years old		65–74 years old		75–84 years old		
	*n* (%)	7,845		2,022	(25.8)	1,488	(19.0)	2,378	(30.3)	1,957	(24.9)	
**Demographics**												
Age, years	Mean (SD)	73.6	(5.5)	69.4	(2.7)	78.9	(2.8)	69.5	(2.6)	79.1	(2.8)	—
Years of residence in the neighbourhood, ​ *n* (%)	<10	144	(1.9)	46	(2.3)	23	(1.6)	45	(1.9)	30	(1.6)	^*^
	10–29	299	(3.9)	89	(4.5)	54	(3.7)	101	(4.3)	55	(2.9)	
	30–59	2,406	(31.4)	478	(24.2)	392	(27.2)	924	(39.4)	612	(32.3)	
	>59	4,816	(62.8)	1,366	(69.0)	974	(67.5)	1,277	(54.4)	1,199	(63.2)	
Marital status, *n* (%)	Married	5,363	(71.3)	1,615	(82.3)	1,186	(84.4)	1,650	(71.0)	912	(49.8)	^*^
	Divorced	331	(4.4)	100	(5.1)	38	(2.7)	153	(6.6)	40	(2.2)	
	Widowed	1,552	(20.6)	113	(5.8)	147	(10.5)	458	(19.7)	834	(45.5)	
	Never married	278	(3.7)	135	(6.9)	35	(2.5)	63	(2.7)	45	(2.5)	
Living alone	Yes, *n* (%)	1,020	(13.3)	235	(11.9)	123	(8.6)	287	(12.2)	375	(19.8)	^*^
**Socio-economic status**												
Years of education, *n* (%)	Other/Unknown	154	(2.0)	30	(1.5)	18	(1.2)	58	(2.5)	48	(2.5)	^*^
	<12	2,964	(38.7)	732	(36.9)	710	(49.2)	603	(25.8)	919	(48.7)	
	≥12	4,532	(59.2)	1,223	(61.6)	714	(49.6)	1,673	(71.7)	922	(48.8)	
Taxation status, *n* (%)	Tax-free	2,103	(26.8)	346	(17.1)	374	(25.1)	650	(27.3)	733	(37.5)	^*^
	Tax-free, but cohabitant is taxed	2,661	(33.9)	236	(11.7)	262	(17.6)	1,150	(48.4)	1,013	(51.8)	
	Taxed, <1.2 million yen	1,397	(17.8)	479	(23.7)	440	(29.6)	360	(15.1)	118	(6.0)	
	Taxed, ≥1.2 million yen	1,684	(21.5)	961	(47.5)	412	(27.7)	218	(9.2)	93	(4.8)	
Current job, *n* (%)	Full-time	1,321	(17.9)	683	(34.7)	186	(13.2)	325	(14.3)	127	(7.3)	^*^
	Part-time	1,612	(21.8)	500	(25.4)	330	(23.5)	492	(21.7)	290	(16.6)	
	Unemployed	4,449	(60.3)	783	(39.8)	891	(63.3)	1,449	(63.9)	1,326	(76.1)	
**Medical and lifestyle profiles**												
Drinking habits, *n* (%)	Current	2,820	(36.3)	1,349	(67.0)	765	(52.1)	505	(21.4)	201	(10.5)	^*^
	Former	1,102	(14.2)	337	(16.7)	387	(26.3)	224	(9.5)	154	(8.0)	
	Never	3,837	(49.5)	327	(16.2)	317	(21.6)	1,627	(69.1)	1,566	(81.5)	
Smoking status, *n* (%)	Current	837	(10.8)	510	(25.3)	198	(13.5)	93	(3.9)	36	(1.9)	^*^
	Former	2,267	(29.2)	1,111	(55.2)	867	(59.0)	198	(8.4)	91	(4.7)	
	Never	4,656	(60.0)	392	(19.5)	405	(27.6)	2,067	(87.7)	1,792	(93.4)	
Self-rated health	Excellent to good, *n* (%)	5,288	(72.5)	1,424	(75.1)	905	(66.6)	1,752	(77.9)	1,207	(67.2)	^*^
Number of chronic diseases, *n* (%)	0	929	(12.1)	271	(13.6)	144	(9.9)	344	(14.7)	170	(8.9)	^*^
	1	2,049	(26.7)	531	(26.7)	372	(25.7)	667	(28.5)	479	(25.1)	
	≥2	4,704	(61.2)	1,188	(59.7)	934	(64.4)	1,326	(56.7)	1,256	(65.9)	
BMI (kg/m^2^), *n* (%)	<18.5	402	(5.2)	49	(2.4)	62	(4.3)	141	(6.0)	150	(8.0)	^*^
	18.5–24.9	5,087	(66.3)	1,333	(66.5)	960	(66.8)	1,573	(67.0)	1,221	(64.8)	
	≥25.0	2,185	(28.5)	623	(31.1)	416	(28.9)	634	(27.0)	512	(27.2)	
Musculoskeletal pain	Shoulder, yes, *n* (%)	1,438	(21.4)	389	(21.1)	285	(22.8)	393	(18.6)	371	(24.6)	^*^
	Lower back, yes, *n* (%)	2,204	(31.1)	518	(27.0)	451	(34.0)	578	(26.5)	657	(39.7)	^*^
	Knee, yes, *n* (%)	1,975	(27.8)	367	(19.6)	358	(27.5)	584	(26.2)	666	(39.3)	^*^
Mobility limitation	With, *n* (%)	2,619	(34.1)	373	(18.8)	547	(38.0)	639	(27.2)	1,060	(55.3)	^*^
Engaging in any exercise more than once ​ a week	Yes, *n* (%)	5,034	(65.9)	1,215	(60.8)	1,017	(70.6)	1,490	(63.9)	1,312	(70.1)	^*^
Sitting time	Hours, mean (SD)	5.8	(3.9)	5.4	(3.9)	5.7	(3.9)	5.8	(3.8)	6.3	(4.0)	^*^
DVS	Score, mean (SD)	3.8	(2.4)	3.1	(2.3)	3.8	(2.4)	4.0	(2.4)	4.4	(2.4)	^*^
	Score ≥4, *n* (%)	3,674	(51.5)	739	(39.2)	656	(49.5)	1,223	(55.3)	1,056	(61.8)	^*^
Eating alone	Mainly eats alone, *n* (%)	969	(12.4)	274	(13.6)	153	(10.4)	216	(9.1)	326	(16.7)	^*^
	Times eating alone per week, mean (SD)	1.6	(2.6)	1.6	(2.6)	1.3	(2.4)	1.4	(2.5)	2.0	(2.9)	^*^
Difficulty falling asleep (≥30)	With, *n* (%)	3,318	(44.5)	705	(35.9)	647	(45.9)	989	(43.3)	977	(54.4)	^*^
Quality of sleep	Excellent to good, *n* (%)	5,677	(73.4)	1,476	(73.5)	1,125	(76.9)	1,706	(72.5)	1,370	(71.5)	^*^
TMIG-IC (0–13)	Score ≥11, *n* (%)	5,934	(80.1)	1,451	(74.7)	945	(68.9)	2,080	(90.5)	1,458	(81.2)	^*^
**GEJE-related items**												
Number of relocations, *n* (%)	0	5,333	(69.3)	1,411	(70.7)	1,017	(70.0)	1,627	(69.1)	1,278	(67.5)	^*^
	1	715	(9.3)	199	(10.0)	156	(10.7)	201	(8.5)	159	(8.4)	
	2	760	(9.9)	201	(10.1)	125	(8.6)	233	(9.9)	201	(10.6)	
	≥3	888	(11.5)	186	(9.3)	154	(10.6)	292	(12.4)	256	(13.5)	
Current home, *n* (%)	Same as before disaster	5,344	(69.1)	1,420	(71.0)	1,027	(70.2)	1,635	(69.5)	1,262	(65.9)	^*^
	Disaster public housing complex	224	(2.9)	66	(3.3)	42	(2.9)	72	(3.1)	44	(2.3)	
	Group relocated housing complex	1,061	(13.7)	255	(12.8)	189	(12.9)	328	(13.9)	289	(15.1)	
	Rented house	573	(7.4)	113	(5.7)	107	(7.3)	163	(6.9)	190	(9.9)	
	Independent reconstruction house	303	(3.9)	84	(4.2)	51	(3.5)	89	(3.8)	79	(4.1)	
	Other	226	(2.9)	62	(3.1)	46	(3.1)	66	(2.8)	52	(2.7)	
Change in consultation access, *n* (%)	Positive change	757	(10.1)	134	(6.9)	121	(8.6)	248	(10.8)	254	(13.9)	^*^
	Negative change	1,774	(23.7)	422	(21.6)	392	(27.8)	441	(19.3)	519	(28.4)	
	Unchanged	4,942	(66.1)	1,395	(71.5)	895	(63.6)	1,600	(69.9)	1,052	(57.6)	

BMI, body mass index; TMIG-IC, Tokyo Metropolitan Institute of Gerontology Index of Competence.

Note: Missing values were removed; *P*-value for the difference among four groups: ^*^P < 0.001.

Table 4 shows frailty and psychological and social function baseline characteristics. Overall, 23.5% of participants were frail; the ratio was higher among people aged 75 or over regardless of sex. Regarding psychological function, depressive moods, apathy, and trauma were more frequent in women aged 75 or older. Women were more anxious than men. Loneliness was most prevalent among men aged 75 or older. Regarding social function, 29.1% of participants had no contact with others apart from their cohabitating family each week and 37.0% participated in social activities at least once a month. Men tended to be isolated and to have low levels of social activities. However, there was no sex difference in social capital.

**Table 4.** Participants’ baseline characteristics: frailty and psychological and social function

		Total		Men				Women				*P*
				65–74 years old		75–84 years old		65–74 years old		75–84 years old		
	*n* (%)	7,845		2,022	(25.8)	1,488	(19.0)	2,378	(30.3)	1,957	(24.9)	
**Primary outcome**												
**Frailty**												
CL15 (0–15)	Mean (SD)	2.3	(2.1)	2.1	(2.0)	2.6	(2.6)	1.9	(1.8)	2.8	(2.3)	^*^
	Score ≥4, *n* (%)	1,659	(23.5)	379	(20.4)	352	(27.2)	388	(17.5)	540	(32.0)	^*^
**Secondary outcome**												
**Psychological function**												
Current feeling												
Depressive mood	GDS-5D ≥2, *n* (%)	3,147	(42.7)	779	(40.1)	567	(41.1)	985	(43.2)	816	(46.1)	^*^
Apathy	GDS-3A ≥2, *n* (%)	3,487	(46.5)	828	(42.1)	684	(48.6)	1,016	(44.1)	959	(52.9)	^*^
Trauma	With, *n* (%)	2,705	(35.3)	560	(28.1)	474	(33.0)	846	(36.0)	825	(43.6)	^*^
Anxiety	With, *n* (%)	3,442	(45.3)	820	(41.3)	603	(42.4)	1,102	(47.2)	917	(49.6)	^*^
Loneliness	The 3-item UCLA loneliness scale (3–9) ≥6, *n* (%)	1,484	(19.3)	386	(19.4)	319	(22.1)	418	(17.8)	361	(19.2)	0.012
Subjective well-being	WHO-5 Well-Being Index (0–25), mean (SD)	14.9	(6.1)	14.3	(6.2)	14.9	(6.2)	15.0	(6.0)	15.4	(6.0)	^*^
	WHO-5 Well-Being Index ≤12, *n* (%)	2,309	(31.4)	673	(34.7)	433	(31.7)	704	(30.8)	499	(28.4)	^*^
Subjective cognitive decline	With, *n* (%)	2,344	(30.9)	572	(29.0)	554	(39.1)	546	(23.5)	672	(35.8)	^*^
Personality traits	TIPI-J, mean (SD)											
	Extraversion	7.9	(2.4)	7.8	(2.4)	7.7	(2.1)	8.1	(2.5)	8.1	(2.4)	^*^
	Agreeableness	10.4	(2.0)	10.2	(2.1)	10.1	(2.1)	10.7	(1.9)	10.7	(2.0)	^*^
	Conscientiousness	8.6	(2.2)	8.6	(2.3)	8.7	(2.2)	8.5	(2.2)	8.7	(2.3)	0.012
	Emotional stability	7.6	(2.2)	7.3	(2.2)	7.5	(2.1)	7.9	(2.3)	7.8	(2.3)	^*^
	Openness	7.3	(2.2)	7.7	(2.3)	7.7	(2.2)	7.0	(2.2)	7.1	(2.2)	^*^
**Social function**												
Social network												
Social isolation	With, *n* (%)	2,213	(29.1)	782	(39.7)	505	(35.6)	538	(23.0)	388	(20.7)	^*^
Social participation	Yes, *n* (%)											
	More than once a month for the following activities:	2,649	(37.0)	614	(32.0)	473	(35.2)	881	(39.6)	681	(40.5)	^*^
	Volunteer groups	749	(10.5)	187	(9.8)	148	(11.0)	238	(10.7)	176	(10.5)	0.657
	Sports groups	1,003	(14)	197	(10.3)	229	(17.1)	332	(14.9)	245	(14.6)	^*^
	Hobby groups	1,178	(16.4)	214	(11.2)	143	(10.7)	495	(22.3)	326	(19.4)	^*^
	Senior citizen clubs	410	(5.7)	43	(2.2)	80	(6.0)	103	(4.6)	184	(10.9)	^*^
	Neighbourhood associations	935	(13.1)	284	(14.8)	173	(12.9)	265	(11.9)	213	(12.7)	0.045
	Salon activities	485	(6.8)	58	(3.0)	66	(4.9)	159	(7.2)	202	(12.0)	^*^
Social support	Receiving instrumental support, with, *n* (%)	5,700	(74.3)	1,477	(73.9)	1,020	(70.9)	1,833	(77.8)	1,370	(73.0)	^*^
	Receiving informational support, with, *n* (%)	6,688	(87.1)	1,680	(84.1)	1,187	(82.2)	2,142	(91.3)	1,679	(88.9)	^*^
	Receiving emotional support, with, *n* (%)	7,144	(92.6)	1,790	(89.5)	1,273	(87.8)	2,271	(96.4)	1,810	(94.9)	^*^
Conversation with others	Every day, yes, *n* (%)	6,914	(89.4)	1,181	(88.9)	1,219	(83.6)	2,195	(92.8)	1,719	(90.0)	^*^
Conversation with other generations	With teenager, *n* (%)	2,465	(33.6)	586	(30.3)	436	(31.8)	833	(36.7)	610	(34.7)	^*^
	With 20s–40s, *n* (%)	3,352	(45.8)	914	(47.2)	561	(41.1)	1,114	(48.9)	763	(43.8)	^*^
	With 50s–60s, *n* (%)	5,827	(77.7)	1,499	(76.1)	995	(71.4)	1,943	(83.8)	1,390	(76.7)	^*^
	With 70s, *n* (%)	6,363	(83.6)	1,498	(76.3)	1,159	(81.2)	2,030	(86.9)	1,676	(89.0)	^*^
Frequency of outings	Every day, yes, *n* (%)	4,623	(60.1)	1,435	(72.6)	893	(61.5)	1,428	(60.6)	867	(45.4)	^*^
Trust in neighbours	Agree/tend to agree, *n* (%)	6,267	(82.5)	1,625	(82.0)	1,190	(83.6)	1,910	(81.6)	1,542	(83.5)	0.219

GDS, Geriatrics Depression Scale; CL15, Check-List 15; TIPI-J, Ten Item Personality Inventory.

Note: Missing values were removed; *P*-value for the difference among four groups: ^*^P < 0.001.

## DISCUSSION

It was difficult to compare these participants’ baseline profiles with those of other GEJE-affected area studies because the measures used were different. Therefore, we compared our results with another cohort, Ota City in Tokyo (The Ota Genki Senior Project).^39^ In brief, Ota City is one of the urban areas in Tokyo, with a population of over 700,000. The Ota Genki Senior Project was a PAR for developing a prototype of a frailty prevention strategy for an urban area. In contrast, Kesennuma City is a coastal rural area with less than one-tenth of the population of Ota City, but both studies used similar indicators (eTable 2).

There was no significant difference in the prevalence of frailty. Kesennuma’s exercise engagement, social participation, and outing frequency were lower, which may be related to high obesity and mobility restrictions. The proportion of people suffering from depressive moods was higher. Regarding social isolation, the results were not significantly different from the urban area, which was reported to have high social isolation.^40^^,^^41^ However, trust in neighbors was a lot higher, which will be a strength in promoting community intervention.

Following reporting of baseline survey results at community meetings in all 16 districts, subsequent actions have been discussed. To deal with isolation and psychological problems, approaches to individuals who had psychosocial rather than physical difficulties in accessing places for social participation were considered. Our intervention will be based on four pillars: exercise, nutrition, social participation, and improvement of mental health.

Our study’s strengths are as follows. First, our response rate of 86.3% means our survey was extensively conducted among community-dwelling older people. Therefore, selection or response bias possibility should be relatively low. By linking the baseline and follow-up data, we will be able to assess various associations and outcomes reflecting the actual conditions of the source population, including an evaluation of the effects of community-based population approaches to frailty prevention. Second, the baseline survey assessed various measures of psychological and social factors, enabling an exploration of the effects of psychological and social factors on frailty from several perspectives.

Our study has some limitations. First, the self-administered questionnaire may be subject to recall bias. Second, the reproducibility of CL15, which is the main outcome, has not been confirmed. Variables about trauma, anxiety, and some social functions were not adequately validated. Third, we did not obtain information before GEJE; its direct impact cannot, therefore, be considered. Finally, this study does not have a randomized design. However, we plan to conduct follow-up questionnaires and collect four outcomes: mortality, LTCI certification, onset of disabled-dementia, and long-term care costs. By analyzing this information and the baseline surveys, we can assess the influence of psychological and social factors on frailty and evaluate the impact of community-based interventions at the individual level. The results of this study may be applied not only to Kesennuma City but also to rural areas that were damaged by the tsunami in coastal rural areas, which have geographical features similar to Kesennuma City.

## SUPPLEMENTARY MATERIAL

eTable 1. Item wording, coding, and dimension for CL15

eTable 2. Comparison between Kesennuma City and Ota City
