# Supplementary material for: The Kesennuma Study in Miyagi, Japan: Study Design and Baseline Profiles of Participants
Source: J Epidemiol. 2022 Dec 5;32(12):559–66. doi: 10.2188/jea.JE20200599 (PMC9643787; doi:10.2188/jea.JE20200599)
Supplement: Supplementary file 1 [file je-32-559-s001.pdf]

eTable 1. Item wording, coding, and dimension for CL15

Dimension	Wording	Coding
Homeboundness	1. Do you usually stay at home all day long?	Yes = 1, No = 0
	2. How often do you usually go out?	More than once per 2-3 days = 0, Less than once a week = 1
	3. Do you have any hobby?	Yes = 0, No = 1
	4. Do you have neighbors who you can talk closely with?	Yes = 0, No = 1
	5. Besides your neighbors, do you have close friends, families, or relatives who you visit?	Yes = 0, No = 1
Falling	6. Have you experienced a fall in the past year?	Yes = 1, No = 0
	7. Can you walk for 1 km?	Yes = 0, No = 1
	8. Can you see things clearly? (with glasses if necessary)	Without difficulty = 0, With difficulty or can not = 1
	9. Do you often slip or stumble at home?	Yes = 1, No = 0
	10. Do you refrain from going out because of fear of falling?	Yes = 1, No = 0
	11. Have you been hospitalized in the past year?	Yes = 1, No = 0
Lower nutrition	12. Do you have appetite these days?	Yes = 0, No = 1
	13. Do you have any difficulty chewing? (even with a denture)	Yes = 1, No = 0
	14. Have you lost 3 kg or more in the past 6 months?	Yes = 1, No = 0
	15. Do you think you have lost muscle or fat in the past 6 months?	Yes = 1, No = 0

CL15, Check-List 15.

eTable 2. Comparison between Kesennuma City and Ota City

		Kesennuma City Miyagi n=9,754		Ota City Tokyo n=5,692		*p*
Persons returning questionnaire	n (%)	8,150	(83.6)	5,820	(77.6)	*
Participants included in analysis	n (%)	7,845	(80.4)	5,692	(75.9)	*
**Demographic**						
Age	Mean (SD)	73.6	(5.5)	74.3	(5.5)	*
Living alone	Yes, n (%)	1,020	(13.3)	1,242	(22.4)	*
**Frailty**						
CL15 (0-15)	Mean (SD)	2.3	(2.1)	2.4	(2.1)	
	Score ≥4, n (%)	1,659	(23.5)	1,243	(24.1)	
**Medical and lifestyle profiles**						
BMI (kg/m^2), n (%)	<18.5	402	(5.2)	446	(7.9)	
	18.5-24.9	5,087	(66.3)	3,974	(70.8)	*
	≥25.0	2,185	(28.5)	1,191	(21.2)	
Mobility limitation	With, n (%)	2,619	(34.1)	1,697	(30.3)	*
Engaging in any exercise more than once a week	Yes, n (%)	5,034	(65.9)	4,095	(73.9)	*
DVS	Mean (SD)	3.8	(2.4)	3.1	(2.2)	
	Score ≥4, n (%)	3,674	(51.5)	2,009	(39.3)	*
**Psychological function**						
Depressive moods	GDS-5D ≥2, n (%)	3,147	(42.7)	1,889	(35.8)	*
Subjective well-being	WHO-5 Well-Being Index (0-25), mean (SD)	14.9	(6.1)	15.2	(6.0)	
**Social function**						
Outings more than once a day	Yes, n (%)	4,623	(60.1)	4,202	(75.0)	*
Social isolation	With, n (%)	2,213	(29.1)	1,664	(29.9)	
Trust in neighbours	Agree/tend to agree, n (%)	6,267	(82.5)	4,129	(76.3)	*
Social participation	At least one of the social activities, n (%)	2,649	(37.0)	2,141	(43.3)	*

BMI, body mass index; CL15, Check-List 15; GDS, Geriatric Depression Scale; SD, standard deviation; WHO, World Health Organization.

Note: We used data from the control group of the intervention study in Ota City.[32] *p*-value for the difference between two groups, *p<0.01
